# Predictive factors of lymph node metastasis in papillary thyroid cancer

**DOI:** 10.1371/journal.pone.0294594

**Published:** 2023-11-27

**Authors:** Woo Jin Song, In Chan Um, Sa Rang Kwon, Jin Ho Lee, Hye Won Lim, Yong Uk Jeong, Seung Min Chung, Jun Sung Moon, Ji Sung Yoon, Kyu Chang Won, Hyoung Woo Lee

**Affiliations:** 1 College of Medicine, Yeungnam University, Daegu, Korea; 2 Division of Endocrinology and Metabolism, Department of Internal Medicine, Yeungnam University College of Medicine, Daegu, Korea; Hamad Medical Corporation, QATAR

## Abstract

This study aimed to evaluate factors that predict lymph node metastasis (LNM) in papillary thyroid cancer (PTC). This retrospective cross-sectional study compared the demographic, clinical, and ultrasonographic findings of patients with PTC with and without LNM. Subgroup analysis was conducted for micro-PTCs (<1 cm). Among total (*n* = 512; mean age, 47.3 ± 12.7 years) and micro-PTC patients (*n* = 312), 35.7% and 19.6% had LNM, respectively. Younger age, male sex, tumor size, bilaterality, and suspicious ultrasound features of the tumor were associated with LNM. In multiple logistic regression analysis, among all patients, age, tumor size, and extrathyroidal extension were independent risk factors for LNM (all *p*<0.05). In the micro-PTC subgroup, age, extrathyroidal extension, bilaterality of tumor, and presence of autoimmune thyroid disease were independent risk and protective factors for LNM (all *p*<0.05). In the receiver operating characteristic analysis, the accuracy of the multivariable logistic regression model for predicting LNM among all patients and micro-PTC was acceptable (area under the curve = 0.729 and 0.733, respectively). Age, sex, tumor size, and extrathyroidal extension can assist in predicting LNM in PTC patients. Additionally, the bilaterality of tumors and presence of autoimmune thyroid disease can assist in predicting LNM in micro-PTCs.

## Introduction

According to the Korea National Cancer Registry, thyroid cancer is the most frequent malignancy in Korea, where its prevalence fell after 2014, but increased again in 2019 [1]. Papillary thyroid carcinoma (PTC) is the most common subtype of thyroid cancer accounting for 80–85% of all thyroid cancers [2, 3]. The prognosis of PTC is generally good, but cervical lymph node metastasis (LNM) is frequently present in up to 50–60% of cases at the time of diagnosis, therefore, prophylactic lymph node dissection is generally performed with total thyroidectomy [4, 5]. Unlike PTC, there is some controversy about the appropriate treatment range for micro-PTC (tumor size <1 cm in maximal diameter) [2], but there is an opinion that prophylactic lymphadenectomy should be performed for micro-PTC as well as PTC [6]. However, owing to such prophylactic lymphadenectomy, even lymph nodes that have not metastasized are often resected. As a result, the extent of the operation is unnecessarily increased and problems such as hypocalcemia and recurrent laryngeal nerve damage occur; therefore controversy about the effectiveness and necessity of prophylactic lymphadenectomy has continued [7]. Therefore, if the required extent of the operation can be determined by predicting LNM before surgery, patients with thyroid cancer can be classified in greater detail, the optimal surgical method can be selected for each patient, and various side effects associated with prophylactic lymphadenectomy can be reduced [8].

Many studies have been conducted on predictive factors of LNM, because LNM in thyroid cancer affects the scope of surgery [9–11]. However, related studies from Korea are limited. Therefore, we aimed to analyze the anthropometric, laboratory, and ultrasonographic (US) risk factors predicting LNM of PTC and micro-PTC.

## Materials and methods

### 1. Patients

**Fig 1:**
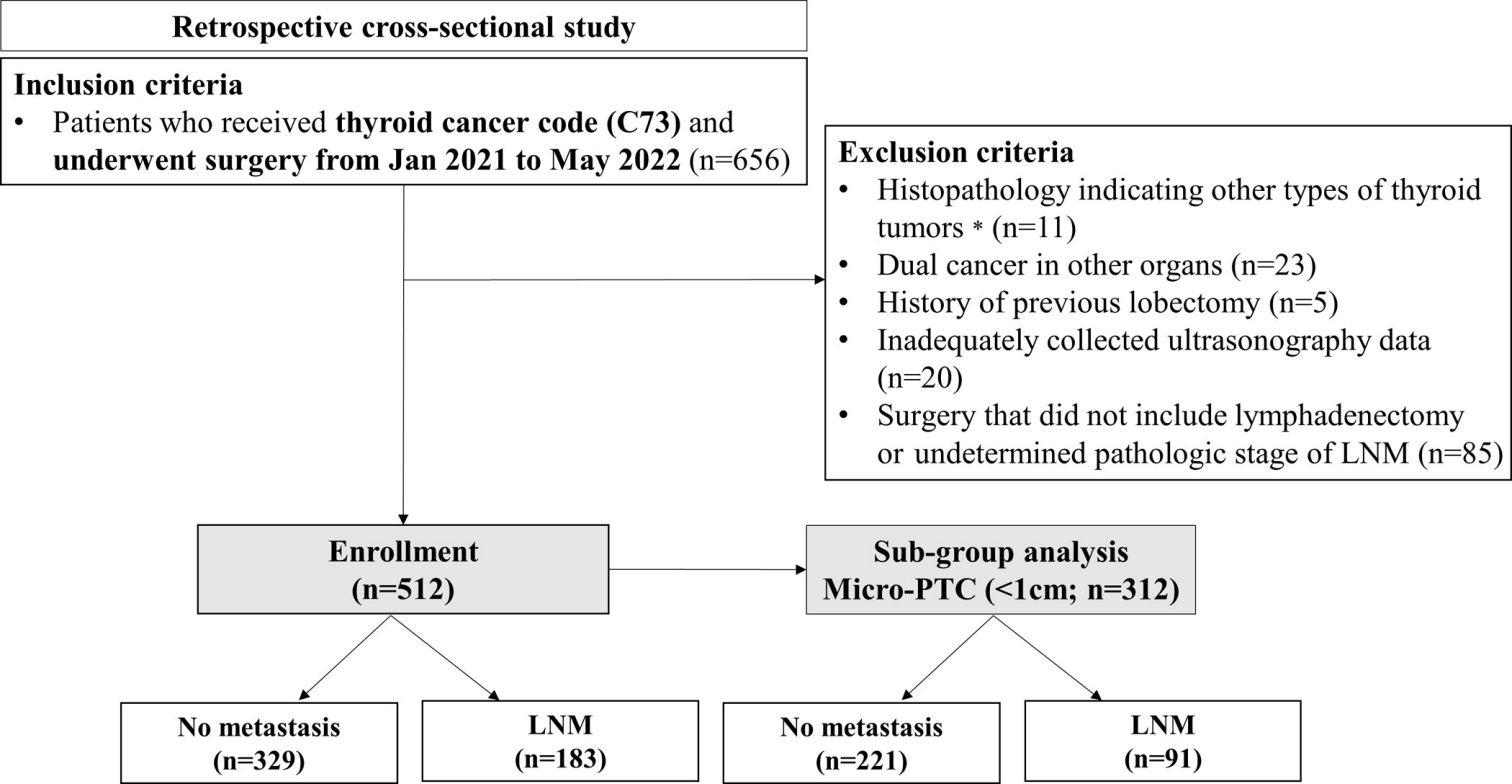
Flow chart of patient selection. * Follicular thyroid cancer (n = 6), medullary thyroid cancer (n = 2), or benign nodules (n = 3). LNM, lymph node metastasis.

This retrospective cross-sectional study screened 656 patients who received a thyroid cancer code (C73) and had undergone thyroid surgery between January 2021 and May 2022 at Yeungnam University Hospital. The exclusion criteria were as follows: 1) histopathology indicating other types of thyroid tumors (*n* = 11); 2) dual cancer in other organs (*n* = 23); 3) history of previous thyroid lobectomy; (*n* = 5); 4) inadequately collected ultrasonography (US) data (*n* = 20); and 5) surgery that did not include lymphadenectomy or undetermined pathologic stage of LNM (*n* = 85). Subsequently, a total of 512 patients were included in the final analysis. Demographic, clinical, and ultrasonographic data were compared between the two groups: LNM group (*n* = 183) and no metastasis group (*n* = 329). The same comparison was also conducted in the micro-PTC subgroup (*n* = 312): the LNM group (*n* = 91) and the no metastasis group (*n* = 221) (**Fig 1**). This study was approved and exempted from informed consent by the institutional review board of Yeungnam University Hospital (IRB No. 2023-01-011). All methods were carried out in accordance with relevant guidelines and regulations.

### 2. Data collection

We have accessed the medical records of Yeungnam University Hospital from January 15, 2023, after IRB approval. All data collection was conducted from January 15, 2023, to January 22, 2023.

Demographic and clinical data of the patients were collected, including age, sex, body mass index (BMI, kg/m^2^), familial history of thyroid cancer, presence of autoimmune thyroid disease (histologically proven Hashimoto’s thyroiditis or chronic lymphocytic thyroiditis); and the laboratory data collected included level of thyroid stimulating hormone (TSH), free thyroxine (free T4), total triiodothyronine (total T3), thyroglobulin, and thyroglobulin antibody (Ab). Thyroglobulin Ab was considered positive if the level was > 60 IU/mL. Thyroid hormone results were obtained by a standard procedure using a radioimmunoassay (KaiEn Gamma Pro, Hoil BioMed Co., South Korea; RALS Gamma Counter, Shin Jin Medics Inc., South Korea).

All US images were retrospectively reviewed and cross-checked by two physicians. Ultrasonographic findings of thyroid nodules were evaluated using the 2021 Korean Thyroid Imaging Reporting and Data System (K-TIRADS) [12]. The following sonographic findings were collected: multifocality (single/multifocal), bilaterality (unilateral/bilateral), size (continuous[cm] and categorical [<1 cm/ ≥1 cm]), composition (cystic or spongiform/predominantly cystic/predominantly solid/solid), echogenicity (hypoechoic/ iso-hyperechoic), orientation (parallel/ nonparallel [taller-than-wide]), margin (smooth/irregular), calcification (absent/microcalcification/macrocalcification), and extrathyroidal extension (absent/present). In addition, the risk of malignancy of thyroid nodules were classified into K-TIRADS 4 (intermediate suspicion) and 5 (high suspicion) based on the composition, echogenicity, orientation, margin, and calcification of the nodule.

### 3. Outcome

The outcome was designated as LNM. Assessment of LNM was based on postoperative biopsy, with a specimen acquired from central compartment neck dissection (CCND) or modified radical neck dissection (MRND). All pathological findings were confirmed by experienced pathologists.

### 4. Sample size estimation

The sample size estimation for LNM was performed using previously published data. A previous prospective study demonstrated that among 184 patients with unilateral PTC who underwent total thyroidectomy and bilateral central lymph node dissection, ipsilateral LNM was reported in 42.9% of the cases [13]. We projected a sample size of 364 to obtain a power of 0.80 with an alpha of 0.05. Therefore, our study had adequate power to detect LNM in patients with PTC.

### 5. Statistical analysis

The χ^2^ test was used for categorical variables and the independent t-test for continuous variables. Continuous variables are expressed as mean ± standard deviation and categorical variables as numbers (percentages). Multivariable logistic regression analysis with backward stepwise elimination method was performed to determine risk factors of LNM. Odds ratios (ORs) and 95% confidence intervals (Cis) were also calculated. Receiver-operating characteristic (ROC) analysis and comparison of independent ROC curves were performed to assess the clinical prediction model for LNM. A value of *p*< 0.05 was considered to be of statistical significance. All statistical analyses was done using IBM SPSS version 19.0 (SPSS Inc., Chicago, IL, USA) and R software (version 3.6.3, R Foundation, Vienna, Austria).

## Results

### 1. Baseline characteristics

The patients were divided into groups: with (*n* = 183) and without LNM (*n* = 329), and their baseline characteristics are summarized in **Table 1**. Patients underwent unilateral thyroidectomy (*n* = 369) or total thyroidectomy (*n* = 143), and either MRND (*n* = 28), or CCND (*n* = 484). Compared to patients without metastasis, those with LNM were predominantly male (26.8% vs. 18.8%, *p* = 0.037), younger (43.41 ± 13.49 vs. 49.34 ± 12.71, *p* < 0.001), and had higher levels of thyroglobulin (21.75 ± 36.90 vs. 14.71 ± 25.95, *p* = 0.029).

**Table 1:** Baseline characteristics of total patients.

	Total (n = 512)	No metastasis (n = 329)	Lymph node metastasis (n = 183)	p-value
**Sex**				
Male, n(%)	111 (21.7%)	62 (18.8%)	49 (26.8%)	0.037
Female, n(%)	401 (78.3%)	267 (81.2%)	134 (73.2%)	
**Age, years**	47.34±12.71	49.53±11.70	43.41±13.49	<0.001
**BMI, kg/m** ^**2**^	24.80±3.92	24.77±3.71	24.84±4.25	0.849
**Familial history of thyroid cancer, n(%)**	62 (13.1%)	39 (11.9%)	23 (12.6%)	0.812
**Autoimmune thyroid disease, n(%)**	146 (28.5%)	101 (30.7%)	45 (24.6%)	0.172
**Laboratory data**				
TSH, mIU/L	2.49±2.73	2.52±3.20	2.43±1.58	0.714
Free T4, pmol/L	15.46±3.55	15.53±3.44	15.33±3.74	0.530
Total T3, nmol/L	1.71±0.45	1.72±0.48	1.68±0.37	0.327
Thyroglobulin, ng/mL ^*^	17.18±30.40	14.71±25.95	21.75±36.90	0.029
Thyroglobulin Ab positivity, n(%)^§^	71 (16.1%)	53 (18.2%)	18 (12.0%)	0.093
Thyroglobulin Ab, IU/mL ^§^	247.3±453.7	174.2±287.1	458.5±724.3	0.121

^*^ 30 and

^§^ 72 data missing; Continuous variables are expressed as mean ± standard deviation, and categorical variables as numbers (percentages).

BMI, body mass index; TSH, thyroid stimulating hormone

Patients with micro-PTC were divided into groups: with (*n* = 91) and without LNM (*n* = 221), and their baseline characteristics are summarized in **S1 Table in S1 File**. Compared to patients without metastasis, those with LNM were predominantly male (25.3% vs. 14.5%, *p* = 0.023) and younger (44.04 ± 11.08 vs. 48.97 ± 11.12, *p* < 0.001). In addition, the number of patients with autoimmune thyroid disease (18.7% vs. 30.3%, *p* = 0.049*)* and positive thyroglobulin antibodies (9.0% vs. 19.2%, *p* = 0.039) was lower in those with LNM compared to those without metastases.

### 2. Sonographic findings

The sonographic findings of the thyroid nodules and their comparison according to LNM are summarized in **Table 2**. Nodules with the following features were more commonly found in patients with LNM than in those without metastases: bilaterality (25.1% vs. 16.7%, *p* = 0.022), larger size (≥1 cm; 50.3% vs. 32.8%, *p* < 0.001), predominantly solid or solid composition (*p* = 0.002), irregular margins (97.3% vs. 92.1%, *p* = 0.019), microcalcification (27.9% vs. 14.3%, *p* < 0.001) or macrocalcification(11.5% vs. 7.6%, *p* < 0.001), and extrathyroidal extension (53.0% vs. 27.1%, *p* < 0.001). In contrast, multifocality, echogenicity, orientation, and K-TIRADS did not show significant association with LNM.

**Table 2:** Sonographic findings of thyroid nodules of total patients.

	Total (n = 512)	No metastasis (n = 329)	Lymph node metastasis (n = 183)	p-value
**Multifocality**				
Single, n(%)	336 (65.6%)	226 (68.7%)	110 (60.1%)	0.050
Multifocal, n(%)	176 (34.3%)	103 (31.3%)	73 (39.9%)	
**Bilaterality**				
Unilateral, n(%)	411 (80.3%)	274 (83.3%)	137 (74.9%)	0.022
Bilateral, n(%)	101 (19.7%)	55 (16.7%)	46 (25.1%)	
**Size, cm**	1.11±0.81	0.98±0.70	1.34±0.93	<0.001
<1cm, n(%)	312 (60.9%)	221 (67.2%)	91 (49.7%)	<0.001
≥1cm, n(%)	200 (39.1%)	108 (32.8%)	92 (50.3%)	
**Composition**				
Cystic or spongiform, n(%)	0	0	0	0.002
Predominantly cystic, n(%)	198 (38.7%)	142 (43.2%)	56 (30.6%)	
Predominantly solid, n(%)	202 (39.5%)	112 (34.0%)	90 (49.2%)	
Solid, n(%)	112 (21.9%)	75 (22.8%)	37 (20.2%)	
**Echogenicity**				
Iso-hyperechoic, n(%)	114 (22.3%)	75 (22.8%)	39 (21.3%)	0.699
Hypoechoic, n(%)	398 (77.7%)	254 (77.2%)	144 (78.7%)	
**Orientation**				
Parallel, n(%)	408 (79.7%)	259 (78.7%)	149 (81.4%)	0.467
Nonparallel (taller-than-wide), n(%)	104 (20.3%)	70 (21.3%)	34 (18.6%)	
**Margin**				
Smooth, n(%)	31 (6.1%)	26 (7.9%)	5 (2.7%)	0.019
Irregular, n(%)	481 (93.9%)	303 (92.1%)	178 (97.3%)	
**Calcification** ^*^				
Absent, n(%)	368 (71.9%)	257 (78.1%)	111 (60.7%)	<0.001
Microcalcification, n(%)	98 (19.1%)	47 (14.3%)	51 (27.9%)	
Macrocalcification, n(%)	46 (9.0%)	25 (7.6%)	21 (11.5%)	
**Extrathyroidal extension**				
Absent, n(%)	326 (63.7%)	240 (72.9%)	86 (47.0%)	<0.001
Present, n(%)	186 (36.3%)	89 (27.1%)	97 (53.0%)	
**K-TIRADS** ^†^				
4 Intermediate suspicion, n(%)	136 (26.6%)	95 (28.9%)	41 (22.4%)	0.138
5 High suspicion, n(%)	376 (73.4%)	234 (71.1%)	142 (77.6%)	

^*^ Microcalcification is defined as punctate (≤1 mm) hyperechoic foci within the solid component of a nodule, and macrocalcification is defined as large (>1 mm) hyperechoic foci with posterior acoustic shadowing.

^†^ K- TIRADS5 is defined as a solid hypoechoic nodule with any of the three suspicious US features: nonparallel orientation, irregular margins, and microcalcification.

K-TIRADS4 is defined as 1) Solid hypoechoic nodules without any of the three suspicious US features,or 2) Partially cystic or iso-/hyperechoic nodule with any of the three suspicious US features, or 3) entirely calcified nodules.

Comparisons of the US findings of micro-PTC nodules according to LNM are summarized in **S2 Table in S1 File**. Nodules with the following features were more commonly found in patients with LN metastases than in those without metastases: larger size (0.70 ± 0.17, 0.64 ± 0.18, *p* = 0.006), irregular margins (97.8% vs. 91.4%, *p* = 0.04), and extrathyroidal extension (39.6% vs. 21.3%, *p* = 0.001). In contrast, multifocality, location, composition, echogenicity, orientation, calcification, and K-TIRADS did not show significant association with LNM.

### 3. Analysis of risk factors for LNM

Logistic regression analysis was performed to analyze the independent risk factors related to LNM among all patients and in the micro-PTC subgroup. The following variables were considered: sex, age, autoimmune thyroid disease, TSH, thyroglobulin, thyroglobulin Ab, multifocality, bilaterality, size (≥1 cm vs. <1 cm among total patients), extrathyroidal extension, and K-TIRADS. The significant risk factors were identified using backward stepwise elimination.

Among all patients, in the crude analysis, male sex, age, thyroglobulin, bilaterality, size ≥1 cm, and extrathyroidal extension were found to be risk factors for LNM (**Table 3**). During the six steps of backward stepwise elimination, TSH, multifocality, thyroglobulin Ab, sex, and thyroglobulin were sequentially eliminated. After multivariable adjustment, six variables were identified as significant: age, autoimmune thyroid disease, bilaterality, size, extrathyroidal extension, and K-TIRADS. Among them, age (adjusted OR = 0.95, 95% CI 0.94–0.97, *p* < 0.001), size ≥ 1 cm (adjusted OR = 1.76, 95% CI 1.1–2.79, *p* = 0.017), and extrathyroidal extension (adjusted OR = 2.86, 95% CI 1.81–4.52, *p* < 0.001) were found to be significant risk factors for LNM.

**Table 3:** Risk factors for lymph node metastasis among total patients.

Risk factor	Crude OR (95% CI)	Crude p-value	Adjusted OR^*^ (95% CI)	Adjusted p-value
Men vs. women	1.57 (1.03, 2.42)	0.038		
Age	0.96 (0.95, 0.98)	<0.001	0.95 (0.94, 0.97)	<0.001
Autoimmune thyroid disease	0.74 (0.49, 1.11)	0.143	0.66 (0.4, 1.07)	0.094
TSH	0.99 (0.92, 1.06)	0.715		
Thyroglobulin	1.01 (1, 1.01)	0.021		
Thyroglobulin Ab	1 (1, 1)	0.259		
Multifocal vs. Single	1.46 (1, 2.12)	0.051		
Bilateral vs. unilateral	1.67 (1.08, 2.6)	0.023	1.68 (0.98, 2.87)	0.058
Size ≥ 1 cm vs. size < 1 cm	2.07 (1.43, 3)	<0.001	1.76 (1.1, 2.79)	0.017
Extrathyroidal extension	3.04 (2.08, 4.44)	<0.001	2.86 (1.81, 4.52)	<0.001
K-TIRADs: 5 vs. 4	1.41 (0.92, 2.14)	0.133	1.62 (0.95, 2.77)	0.078

^*^Backward stepwise elimination.

TSH, thyroid stimulating hormone, OR, odds ratio; CI, confidence interval

K-TIRADS: K- TIRADS5 is defined as a solid hypoechoic nodule with any of the three suspicious US features: nonparallel orientation, irregular margins, and microcalcification.

K-TIRADS4 is defined as 1) solid hypoechoic nodules without any of the three suspicious US features, 2) partially cystic or iso-/hyperechoic nodules with any of the three suspicious US features, or 3) entirely calcified nodules.

Among the micro-PTC subgroup, in the crude analysis, male sex, age, autoimmune thyroid disease, bilaterality, and extrathyroidal extension were found to be risk factors for LNM (**Table 4**). During seven steps of backward stepwise elimination, K-TIRADS, multifocality, TSH, thyroglobulin, sex, and thyroglobulin Ab were sequentially eliminated. After multivariable adjustment, four variables were identified to be significant: age (adjusted OR = 0.95, 95% CI 0.93–0.98, *p* < 0.001), bilaterality (adjusted OR = 2.23, 95% CI 1.08–4.62, *p* = 0.03), extrathyroidal extension (adjusted OR = 2.5, 95% CI 1.37–4.59, *p* = 0.003) were found to be significant risk factors for LNM. In contrast, autoimmune thyroid disease (adjusted OR = 0.47, 95% CI 0.24–0.94, *p* = 0.031) was found to be a significant protective factor for LNM.

**Table 4:** Risk factors for lymph node metastasis among micro-PTC subgroups.

Risk factor	Crude OR (95% CI)	Crude p-value	Adjusted OR^*^ (95% CI)	Adjusted p-value
Men vs. women	2 (1.09, 3.65)	0.025		
Age	0.96 (0.94, 0.98)	<0.001	0.95 (0.93, 0.98)	<0.001
Autoimmune thyroid disease	0.53 (0.29, 0.96)	0.037	0.47 (0.24, 0.94)	0.031
TSH	0.89 (0.76, 1.06)	0.199		
Thyroglobulin	1 (1, 1.01)	0.354		
Thyroglobulin Ab	1 (0.99, 1)	0.342		
Multifocal vs. Single	1.49 (0.88, 2.51)	0.136		
Bilateral vs. unilateral	1.82 (0.96, 3.46)	0.068	2.23 (1.08, 4.62)	0.03
Extrathyroidal extension	2.42 (1.43, 4.11)	0.001	2.5 (1.37, 4.59)	0.003
K-TIRADs: 5 vs. 4	1.75 (0.9, 3.42)	0.098		

^*^Backward stepwise elimination.

TSH, thyroid stimulating hormone, OR, odds ratio; CI, confidence interval

K-TIRADS: K- TIRADS5 is defined as a solid hypoechoic nodule with any of the three suspicious US features: nonparallel orientation, irregular margins, and microcalcification.

K-TIRADS4 is defined as 1) solid hypoechoic nodules without any of the three suspicious US features, 2) partially cystic or iso-/hyperechoic nodules with any of the three suspicious US features, or 3) entirely calcified nodules.

### 4. Predictability of factors for LNM

**Fig 2:**
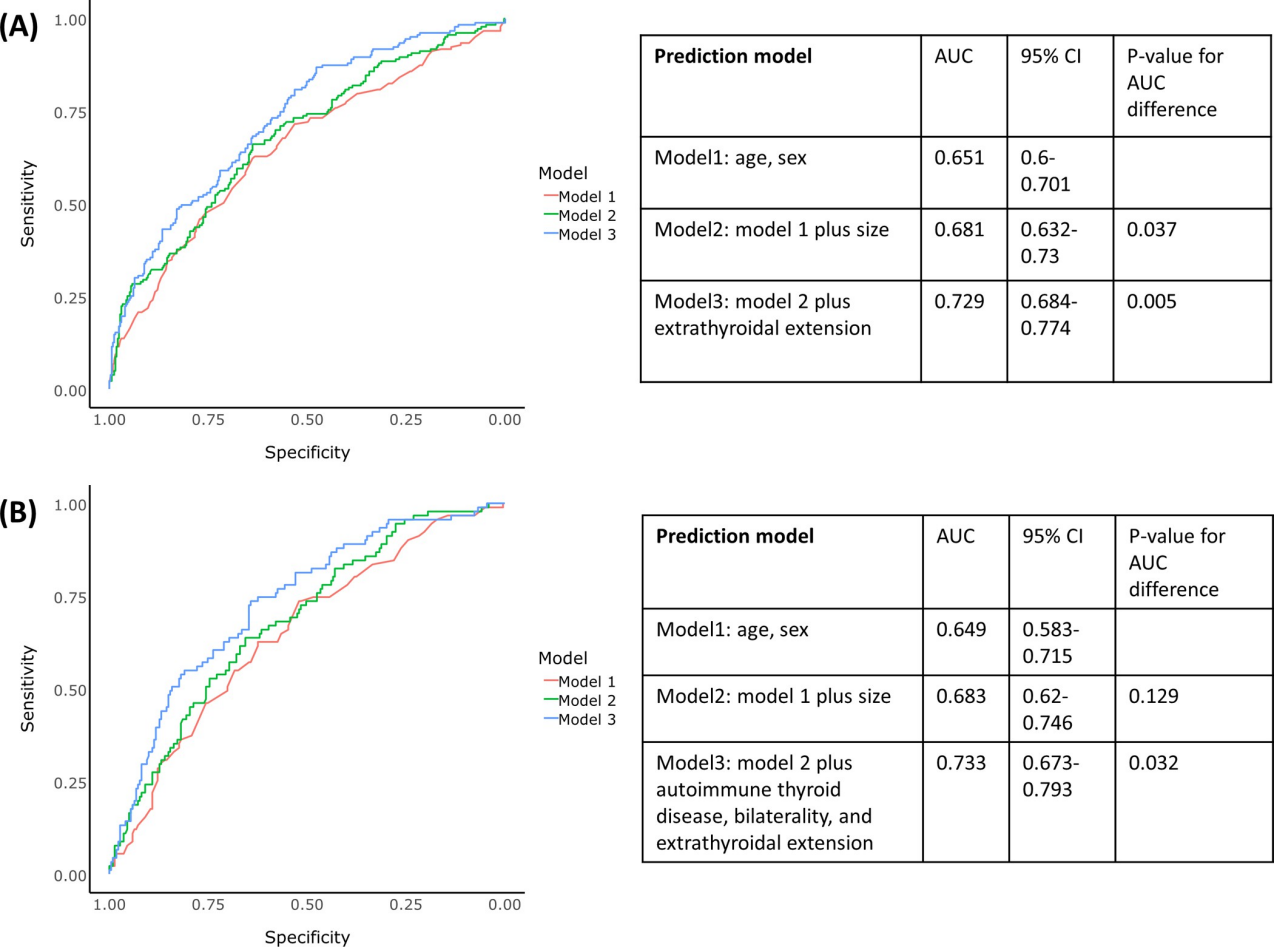
Predictability of factors for lymph node metastasis among (A) total patients and (B) Micro-PTC (< 1cm).

Multivariable regression analysis, revealed the significant risk factors for LNM of PTC to be age, size, and extrathyroidal extension. The prediction accuracy of the multivariable logistic regression models for LNM was calculated using ROC curve analysis (**Fig 2A**). Model 1 included age and sex; model 2 included age, sex, and size; and model 3 included age, sex, size, and extrathyroidal extension. Model 2 had a significantly higher prediction accuracy of LNM than models 1, and model 3 than model 2 (*p* < 0.05, both). In particular, the prediction accuracy (area under curve, AUC) of model 3 was 0.729 (95% CI = 0.684–0.774), which was an acceptable level.

In the multivariable regression analysis, the significant risk factors for LNM of micro-PTC were age, autoimmune thyroid disease, bilaterality, and extrathyroidal extension. The prediction accuracy of these factors for LNM was calculated using the ROC curve analysis (**Fig 2B**). Model 1 included age and sex, model 2 included age, sex, and size and model 3 included age, sex, size, autoimmune thyroid disease, bilaterality, and extrathyroidal extension. The prediction accuracy of LNM was comparable between models 1 and 2 (*p* = 0.129), however, model 3 had a significantly higher prediction accuracy for LNM than model 2 (*p* < 0.05). In particular, the prediction accuracy (area under the curve, AUC) of model 3 was 0.733 (95% CI = 0.673–0.793), which is an acceptable level.

## Discussion

This study explored the risk factors that can predict LNM in PTC and micro-PTC patients. Male sex, younger age, bilaterality, large tumor size, and US features suggesting malignancy were associated with LNM. After multivariable adjustment with backward stepwise elimination, age, size ≥1 cm, and extrathyroidal extension were found to be significant risk factors for LNM in PTC. In addition, age, bilaterality, and extrathyroidal extension were significant risk factors, and autoimmune thyroid disease appeared to be a protective factor for LNM among micro-PTCs.

The initial therapy for PTC is the surgical removal of the primary tumor and clinically significant LNM [14]. In addition to therapeutic lymph node dissection for cN1 disease, prophylactic lymphadenectomy is generally performed to reduce metastasis or local recurrence, however, this practice remains debatable [6, 15, 16]. Owing to such prophylactic lymphadenectomy, even lymph nodes that have not been metastasized are often resected. As a result, the range of surgery is unnecessarily increased and problems such as hypocalcemia and recurrent laryngeal nerve damage can occur [7]. Active surveillance or image-guided thermal ablation is emerging as an alternative treatment modality for low-risk micro-PTC [17, 18]. However, LNM may progress, resulting in delayed extensive surgery if clinically LNM is not diagnosed initially [19]. Therefore, if LNM can be predicted at the time of the initial diagnosis, it is expected to reduce unnecessary lymph node dissection and related complications [8]. Although various studies have been conducted on factors that can predict LNM of PTC, there have been controversies, as there are cases in which contradictory results are shown for the same factors.

In this study, age and extrathyroidal extension are found to be common risk factors for LNM in PTC and micro-PTC. Previous studies have demonstrated that the risk of LNM was high for those under the age of 45 in PTC [9] and those under the age of 40 in micro-PTC [20]. These results are consistent with those from this study in that younger age was analyzed as a risk factor. Extrathyroidal extension is reported to be a factor that increases the risk of LNM in patients with PTC and micro-PTC [21–23]. In this study, the presence of extrathyroidal extension increased the risk of LNM 2.86 times in PTC and 2.5 times in micro-PTC.

Previous studies have demonstrated that PTCs larger than 1 cm increase the risk of LNM [12, 24, 25]. In this study, the risk of LNM in PTC larger than 1 cm was 1.76 times higher than that in micro-PTCs. In The ROC analysis, the predictability of LNM in PTC was acceptable in model 3 which included age, sex, tumor size, and extrathyroidal extension. In the case of the aforementioned risk factors, similar results were shown in this study as factors that have been proven to increase the risk of lymphatic metastasis in previous studies [9, 26, 27]. However, among micro-PTCs, there was no difference in the risk of LNM according to size [28]. This was also consistent with this study; in the ROC analysis, the predictability for LNM in micro-PTC was not different between prediction model 1(age, sex) and model 2(model 2 plus size).

Bilaterality and autoimmune thyroid disease are additional predictive factors for LNM in micro-PTCs. In our study bilaterality has shown different results compared with the results of previous studies. Yang et al. found that bilaterality was a significant factor that increases the risk of LNM 1.4 times, but Kim et al. found that bilaterality did not show a significant relationship with LNM [29, 30]. However, bilaterality increased the risk of LNM 2.23 times in this study. In addition, the relationship between autoimmune thyroid disease and LNM of micro-PTC has shown conflicting results in previous studies. Yin et al. showed that autoimmune disease was a significant factor that doubled the LNM of micro-PTCs, while Qu et al. and Medas et al. showed that autoimmune diseases were not significantly related to the LNM of micro-PTCs [10, 31, 32]. In contrast, in two different studies, Kim et al. and Kim et al. reported that the presence of Hashimoto’s thyroiditis or chronic lymphocytic thyroiditis is a negative predictive factor for LNM, which reduces the recurrence rate [33, 34]. One of the factors influencing these conflicting results may be the different definitions of thyroid autoimmune disease, specifically the criteria for positivity of thyroid autoantibodies and/or histologically confirmed autoimmune thyroid disease. In our study, the presence of histologically proven Hashimoto’s thyroiditis or chronic lymphocytic thyroiditis decreased the risk of LNM by half in patients with micro-PTC. A possible mechanism is the Fas-mediated apoptotic pathway. Fas and Fas-ligand are expressed in the follicular cells in Hashimoto’s thyroiditis, which contributes to thyroid destruction [35]. Additionally, Fas expression is also upregulated in cancerous thyrocytes [36]. Accordingly, in patients with autoimmune thyroid disease, the Fas-mediated apoptotic pathway in PTC may result in a favorable prognosis [37]. Another possible reason is that in cases of autoimmune thyroid disease, the thyroid parenchyma is observed to be heterogeneous and is sometimes mistaken for a high degree of malignancy in the process of interpreting ultrasound images [10, 38].

As the predictability of LNM in micro-PTC significantly increased when the coexistence of autoimmune thyroid disease, bilaterality, and extrathyroidal extension were added to age, sex, and size as predictive factors. We suggest that considering these predictive factors and establishing a scoring system might be beneficial in determining the optimal treatment options: surgery with or without lymphadenectomy or active surveillance. Thus, this approach would allow physicians to optimize treatment outcomes and minimize the risk of unnecessary interventions. However, this study did not derive an exact cutoff value from the ROC curve analysis, and thus was insufficient to provide precise criteria. Therefore, further studies that consider the risk factors for LNM in micro-PTCs are required.

This study has certain limitations. First, it was a single center retrospective study conducted among people from the same ethnic group. Second, the referring hospitals used different US equipment, which may have affected the interpretation of the thyroid nodules. Third, TPO Ab, the most sensitive marker for evaluating autoimmune thyroid disease, was not measured in this study. Lastly, genetic mutations and PTC variants were not considered. However, we selected the most important factors predicting LNM using the backward elimination method [39], and the predictability for LNM gradually increased and was acceptable when the selected risk factors were summed up step by step. In addition, we not only reconfirmed previously known risk factors, but also revealed the protective effect of autoimmune thyroid disease on LNM in micro-PTC. Further larger prospective studies are warranted to increase the preoperative prediction accuracy of LNM and help in the decision to perform prophylactic lymph node dissection.

In conclusion, it was demonstrated that factors including age, sex, tumor size, and extrathyroidal extension increased the predictability of LNM in PTC. In micro-PTC, bilaterality and autoimmune thyroid disease significantly increased predictability along with the predictive factors of LNM in PTC. Therefore, these factors should be considered when predicting the occurrence of LNM in patients with PTC.

## Supporting information

S1 File S1 Table. Baseline characteristics of micro-PTC subgroup and S2 Table. Sonographic findings of thyroid nodules of micro-PTC subgroup.
(DOCX)
