# Supplementary material for: Predictive factors of lymph node metastasis in papillary thyroid cancer
Source: PLoS One. 2023 Nov 27;18(11):e0294594. doi: 10.1371/journal.pone.0294594 (PMC10681174; doi:10.1371/journal.pone.0294594)
Supplement: S1 File — S1 Table. Baseline characteristics of micro-PTC subgroup and S2 Table. Sonographic findings of thyroid nodules of micro-PTC subgroup. (DOCX) [file pone.0294594.s001.docx]

**Supplementary Table 1. Baseline characteristics of micro-PTC subgroup**

|  | **Total (n=312)** | **No metastasis (n=221)** | **Lymph node metastasis**  **(n=91)** | **p-value** |
| --- | --- | --- | --- | --- |
| **Sex** |  |  |  |  |
| Male, n(%) | 55 (17.6%) | 32 (14.5%) | 23 (25.3%) | 0.023 |
| Female, n(%) | 257 (82.4%) | 189 (85.5%) | 68 (74.7%) |  |
| **Age, years** | 47.53±11.32 | 48.97±11.12 | 44.04±11.08 | <0.001 |
| **BMI, kg/m^2^** | 24.36±3.72 | 24.41±3.69 | 24.23±3.77 | 0.692 |
| **Familial history of thyroid cancer, n(%)** | 32 (10.3%) | 20 (9.0 %) | 12 (13.2%) | 0.274 |
| **Autoimmune thyroid disease, n(%)** | 84 (26.9%) | 67 (30.3%) | 17 (18.7%) | 0.049 |
| **Laboratory data** |  |  |  |  |
| TSH, mIU/L | 2.29±1.80 | 2.38±1.94 | 2.08±1.40 | 0.198 |
| Free T4, pmol/L | 15.64±3.41 | 15.68±3.56 | 15.53±3.03 | 0.727 |
| Total T3, nmol/L | 1.72±0.49 | 1.74±0.54 | 1.66±0.32 | 0.18 |
| Thyroglobulin, ng/mL * | 11.88±24.36 | 11.02±22.09 | 13.94±29.09 | 0.34 |
| Thyroglobulin antibody positivity, n(%) ^§^ | 45 (16.3%) | 38 (19.2%) | 7 (9.0%) | 0.039 |
| Thyroglobulin antibody, IU/mL ^§^ | 30.93 ± 142.45 | 36.34 ± 159.48 | 17.21 ± 84.50 | 0.316 |

***** 7 and ^§^ 36 data missing; Continuous variables are expressed as mean ± standard deviation and categorical variables as numbers (percentages).

**Supplementary Table 2. Sonographic findings of thyroid nodules of micro-PTC subgroup**

|  | **Total (n=312)** | **No metastasis (n=221)** | **Lymph node metastasis**  **(n=91)** | **p-value** |
| --- | --- | --- | --- | --- |
| **Multifocality** |  |  |  |  |
| Single, n(%) | 221 (70.8%) | 162 (73.3%) | 59 (64.8%) | 0.135 |
| Multifocal, n(%) | 91 (29.2%) | 59 (26.7%) | 32 (35.2%) |  |
| **Bilaterality** |  |  |  |  |
| Unilateral, n(%) | 265 (84.9%) | 193 (87.3%) | 72 (79.1%) | 0.065 |
| Bilateral, n(%) | 47 (15.1%) | 28 (12.7%) | 19 (20.9%) |  |
| **Size, cm** | 0.66±0.19 | 0.64±0.18 | 0.70±0.17 | 0.006 |
| **Composition** |  |  |  |  |
| Cystic or spongiform, n(%) | 0 (%) | 0 (%) | 0 (%) | 0.445 |
| Predominantly cystic, n(%) | 141 (45.2%) | 104 (47.1%) | 37 (40.7%) |  |
| Predominantly solid, n(%) | 104 (33.3%) | 69 (31.2%) | 35 (38.5%) |  |
| Solid, n(%) | 67 (21.5%) | 48 (21.7%) | 19 (20.9%) |  |
| **Echogenicity** |  |  |  |  |
| Iso-hyperechoic, n(%) | 47 (15.1%) | 35 (15.8%) | 12 (13.2%) | 0.552 |
| Hypoechoic, n(%) | 265 (84.9%) | 186 (84.2%) | 79 (86.8%) |  |
| **Orientation** |  |  |  |  |
| Parallel, n(%) | 231 (74.0%) | 163 (73.8%) | 68 (74.7%) | 0.859 |
| Nonparallel (taller-than-wide) , n(%) | 81 (26.0%) | 58 (26.2%) | 23 (25.3%) |  |
| **Margin** |  |  |  |  |
| Smooth, n(%) | 21 (6.7%) | 19 (8.6%) | 2 (2.2%) | 0.04 |
| Irregular, n(%) | 291 (93.3%) | 202 (91.4%) | 89 (97.8%) |  |
| **Calcification**^*^ |  |  |  |  |
| Absent, n(%) | 252 (80.8%) | 180 (81.4%) | 72 (79.1%) | 0.792 |
| Microcalcification, n(%) | 45 (14.4%) | 30 (13.6%) | 15 (16.5%) |  |
| Macrocalcification, n(%) | 15 (4.8%) | 11 (5.0%) | 4 (4.4%) |  |
| **Extrathyroidal extension** |  |  |  |  |
| Absent, n(%) | 229 (73.4%) | 174 (78.7%) | 55 (60.4%) | 0.001 |
| Present, n(%) | 83 (26.6%) | 47 (21.3%) | 36 (39.6%) |  |
| **K-TIRADS** ^†^ |  |  |  |  |
| 4 Intermediate suspicion, n(%) | 63 (20.2%) | 50 (22.6%) | 13 (14.3%) | 0.130 |
| 5 High suspicion, n(%) | 249 (79.8%) | 171 (77.4%) | 78 (85.7%) |  |

^*^ Microcalcification is defined as punctate (≤1 mm) hyperechoic foci within the solid component of a nodule, and macrocalcification is defined as large (>1 mm) hyperechoic foci with posterior acoustic shadowing.

^†^ K- TIRADS5 is defined as solid hypoechoic nodule with any of the three suspicious US features: nonparallel orientation, irregular margins, and microcalcification.

K-TIRADS4 is defined as 1) Solid hypoechoic nodules without any of the three suspicious US features or 2) Partially cystic or iso-/hyperechoic nodule with any of the three suspicious US features, or 3) entirely calcified nodules.
